# Felt affect in good- and poor-outcome schizophrenia

**DOI:** 10.4103/0019-5545.46070

**Published:** 2005

**Authors:** Anuradha Sovani, Shubha Thatte, C.G. Deshpande

**Affiliations:** * Reader in Clinical Psychology Department of Applied Psychology, University of Mumbai, Mumbai; ** Project Consultant Department of Applied Psychology, University of Mumbai, Mumbai; *** Principal Investigator Department of Applied Psychology, University of Mumbai, Mumbai

**Keywords:** Felt affect, schizophrenia, PANAS

## Abstract

**Background:**

Family members and caregivers may misinterpret blunted affect as a true lack of emotion in patients with schizophrenia.

**Aim:**

To assess felt affect or experienced emotion among low- and high-functioning schizophrenics.

**Methods:**

Two hundred people with schizophrenia were assessed using the Global Assessment of Functioning scale of DSM-IV and the Positive and Negative Affect Schedule (PANAS).

**Results:**

The findings reveal that people with good- and poor-outcome schizophrenia show no significant differences in the emotions experienced, implying that felt affect is comparable regardless of the severity of symptoms in chronic schizophrenia. In fact, low-functioning patients scored a mean (SD) of 46.07 (13.13) on the PANAS, in contrast to a slightly lower scored by high-functioning patients (44.33 [12.03]).

**Conclusion:**

Although patients may show flat affect, and therefore be mistakenly considered withdrawn and apathetic by the observer, they do, in fact, experience as much, or perhaps even more emotion than their higher-functioning counterparts.

## INTRODUCTION

Changes in the experience of affect have historically been described as a key feature of schizophrenia. As early as the beginning of the twentieth century, Kraeplin had recorded the striking and profound damage in the emotional life of the patient.

The DSM-IV lists an affective deficit among patients fulfilling Criteria A of the disorder.^1^ Decreased capacity to experience pleasure and anhedonia are associated features. Andreasen listed the negative symptoms of schizophrenia in which affective deficits, including anhedonia and affective flattening, figured prominently.^2^ In fact, they were considered by her as core negative symptoms. Rado also considered anhedonia a central deficit in schizophrenia and attributed it to a strong genetic basis.^3^

It is also interesting to note that anhedonia or hypohedonia, as discussed by Meehl, has been more in the social realm, vis-a-vis lack of pleasure derived from social interactions.^4^ Later, Chapman *et al*. further explored anhedonia by differentiating it into physical and social types.^5^

Fish reported that by the age of 3 years, about half the disturbed schizophrenia-risk subjects studied by him displayed blunted-detached affect.^6^ They were isolated as children and, as adults, all met the criteria for schizophrenia-spectrum disorders. There seem to be studies from various populations worldwide focusing on either increased levels of negatively valenced emotions, or flattening of emotions in general.

Fenton and McGlashan found flat affect to be negatively correlated with premorbid adjustment during both early and late adolescence.^7^ Using behavioural tasks, Bellack reported that affective flattening was significantly correlated with poor role-play ratings of non-verbal skills, negatively correlated with ratings of current interpersonal relationships, and associated with poorer adjustment in a range of social domains.^8^

One of the biggest controversies in this area is whether paucity of emotional expression in people with schizophrenia reflects impoverished emotional experience. If the latter is true, clearly, the patient will not respond emotionally to social-affiliative cues. However, if he/she is able to respond but unable to express these feelings, the actual emotional experience will be masked.

Kring *et al*. found that patients with affective flattening reported levels of affect equivalent to those experienced by schizophrenics with non-flattened affect.^9^ In some cases, they even exceeded the levels of normal controls.

Similar studies by Berenbaum and Oltmanns (1992) concluded that affective flattening is not related to diminished self-reports of mood.^10^ When patients were actually experiencing emotion, clinical observers underestimated the amount of positive affect that blunted schizophrenic patients were experiencing.

A vast amount of research was done in the 1960s and 1970s on the effects of institutionalization.^11^ It was seen that patients who had been in long-stay institutions showed more flattening of affect. In retrospect, one may now ask whether these patients were truly experiencing low levels of emotion.

The 1980s brought a lot of research on the variable labelled EE or expressed emotion in the family. Vaughn researched the poorer prognosis that seemed to accompany high levels of family EE.^12^ Logically, if high EE brought on relapses, the patients must surely be experiencing emotions with a great deal of clarity, i.e. they were highly receptive to them.

Mueser *et al*. found that although patients with highly critical relatives did not respond to the negative affect with assertiveness, their judgement of the negativity of affective displays did not differ from the normal.^13^

Watson *et al*. have created a scale called the Positive and Negative Affect Schedule (PANAS), for the measurement of a subject's experienced mood or feeling levels.^14^

## METHODS

### Sample

Patients fulfilling the DSM-IV criteria for schizophrenia were selected from four centres: 2 from long-stay regional mental hospitals, 1 from a non-governmental organization (NGO), and 1 from the psychiatry department of a municipal general hospital. Of the initial 234 patients screened, 200 were selected on the basis of the inclusion criteria, which were duration of 5 years of chronicity since onset, presence of informant willing and able to participate in the study, and a minimum of fourth-grade level of education. The exclusion criteria were concurrent diagnosis of organicity, substance use or dependence, and institutionalization for over 6 months.

### Tools

The PANAS was used to assess felt or experienced emotion. The scale requires the respondents to indicate the frequency and intensity with which they had experienced various specific emotions over a period of time. Hence, the PANAS assesses the capacity of the respondent to experience emotions of various types, positive as well as negative.

The Global Assessment of Functioning (GAF) scale from DSM-IV, Axis V, was used. This tool rates psychological, social and occupational functioning on a hypothetical continuum on a scale of 10 to 90, with nine class intervals, each having clear guidelines for scoring. Information was collected from the patient and informant, and a rating was made on the basis of current functioning of the patient.

### Procedure

A brief interview with the patient and caregiver allowed the researchers to complete an assessment on the basis of the DSM-IV criteria for schizophrenia, inclusion and exclusion criteria mentioned above, and the GAF scale.

Instructions were then given for the PANAS among other assessments done as part of a larger project.

The PANAS required patients to decide whether they had felt the listed emotion over the past year. Rating points ranged from 1 (very slight) to 5 (a lot). Nineteen emotions were listed in this manner, and scattered in five parts over the entire assessment protocol. This precaution was taken as completing the ratings on all emotions successively was found to be difficult for the patients during the pilot. During the latter, emotion labels in the PANAS were converted to the local language for ease of understanding.

## RESULTS

The mean PANAS scores and standard deviations (SD) were computed of high- and low-functioning schizophrenics, as determined by their GAF scores (Table 1). The difference between the two groups was not statistically significant.

**Table 1:** Mean scores and standard deviations on the PANAS for low-and high-functioning schizophrenics

	Low-functioning schizophrenics	High-functioning schizophrenics
Mean	46.07	44.33
SD	13.13	12.03

PANAS: Positive and Negative Affect Schedule

Tables 2 and 3 present the demographic distribution of the sample, and the distribution of GAF scores, respectively. The latter were used to categorize the patients into high- and low-functioning groups.

**Table 2:** Demographic distribution of the sample of 200 patients with schizophrenia

	Mean	SD
Age (in years)	36.78	9.65
Age at onset (in years)	26.51	8.29
Duration of illness (in years)	10.51	6.26

The study group comprised 104 men and 96 women.

**Table 3:** Global Assessment of Functioning (GAF) score and sex distribution in the study population

GAF range	Total		Males	Females
10–40	13 (6.5)	Low-functioning	63	49
41–60	99 (49.5)	schizophrenics		
61–80	81 (40.5)	High-functioning	41	47
81–90	7 (3.5)	schizophrenics		
Mean GAF score: 61.04 SD GAF score: 12.37				

Values in parentheses are percentages.

## DISCUSSION

It is clear from the findings presented in Table 1 that in spite of the marked difference in the GAF scores of patients in the two groups, the levels of felt affect are not significantly different. Thus, the level of severity of the illness, or poorer prognosis, seems to be independent of this factor of experienced emotion.

People with severe schizophrenic symptoms are often misunderstood, and seen to be aloof, unaffected by the events occurring around them, and insensitive to the feelings of others, particularly caregivers. This myth could be cleared up on the basis of these findings.

Further, it is interesting that although the difference between the two groups is negligible, the affect felt by the low-functioning group is, in fact, marginally higher (46.07) than that experienced by the high-functioning group (44.33). The dispersion of scores in both the groups is about the same. It is therefore possible that the low-functioning patients were in fact able to feel emotions to a greater extent.

Whether the EE was high, or their sensitivity rendered family interactions to be higher on EE, is a question that still remains unsolved.

The implications of these findings are clear. They provide pointers for the counselling of caregivers of people with schizophrenia. Caregivers need to be cautioned against expressing negative emotions freely in the presence of the patient.

An incidental finding in this study was that a Pearson correlation coefficient computed for scores on the audio-taped comparative fit index (CFI)^12^ and the PANAS scores was negligible (0.03) and non-significant. This finding also shows that no clear trend emerged between family EE and felt affect in schizophrenics.

## CONCLUSION

Family members and caregivers may misinterpret blunted expression of affect as a true lack of emotion. They may feel that the patient is either apathetic or insensitive. They may, in other words, misattribute motives, and hence a distance may be created between the caregiver and the patient, at a time when he or she requires support the most. Getting rid of such misconceptions must be a key component of any community awareness programme for caregivers of people with schizophrenia.
